# Evaluation of Early Prognostic Factors of Mortality in Patients with Acute Pancreatitis: A Retrospective Study

**DOI:** 10.1155/2017/8363561

**Published:** 2017-12-25

**Authors:** Wenzheng Zhang, Jiang Hu, Bihui Yao, Xvsheng Yang, Lei Song, Ting Yin, Lu Liang

**Affiliations:** ^1^Department of General Surgery, Baotou Central Hospital, Baotou, China; ^2^Intensive Care Unit, Baotou Central Hospital, Baotou, China

## Abstract

Early and accurate assessment of severity in acute pancreatitis (AP) is of great importance to provide effective disease management and prevent mortality. In this study, we aim to evaluate early indicators that predict the mortality of AP. We retrospectively analyzed 24-hour clinical characteristics and laboratory data in 166 AP patients recruited between January 2014 and November 2015 in Baotou Central Hospital. In total, 18 patients did not survive the disease. Multivariate logistic regression showed that red cell distribution (RDW) (OR = 2.965, *P* = 0.001) and creatinine (OR = 1.025, *P* = 0.005) were early independent risk factors of AP mortality while albumin (OR = 0.920, *P* = 0.032) levels reduced AP mortality. The corresponding optimal cut-off values were 14.45, 125.5, and 34.95, respectively. The positive predictive values of the AP mortality were 80.1%, 54.5%, and 69.5%. In combined measurement, the area under the curve of RDW, creatinine, and albumin was 0.964 (95% CI: 0.924 to 1.000, *P* < 0.001). RDW ≥ 14.45%, creatinine ≥ 125.5 *μ*mol/l, and albumin ≤ 34.95 g/l indicated a good predictive value for mortality in AP patients with a sensitivity of 100% and specificity of 64.2%. RDW, creatinine, and albumin may serve as early indicators for AP mortality which warrants further clinical investigation.

## 1. Introduction

Acute pancreatitis (AP) is a serious inflammatory condition in the pancreatic tissue that represents one of the leading cause of hospitalization for gastrointestinal disorders in many countries [1]. AP has an annual incidence of 13 to 45 per 100,000 persons and is the fifth leading cause of hospital deaths [2]. The clinical spectrum of AP patients varies widely ranging from mild local pancreas inflammation to severe multiorgan failure. Around 20% of AP patients will experience a severe AP attack, leading to a higher risk of systemic complications, pancreatic necrosis, prolonged hospital stay, and increased mortality [1, 3]. Based on the most recent updated Atlanta classification, AP can be classified into three grades: mild acute pancreatitis (MAP), moderately severe acute pancreatitis (MSAP), and severe acute pancreatitis (SAP) [4]. MAP shows no organ failure, nor local or systemic complications. The patients' symptoms usually disappear within 1 to 2 weeks' of hospitalization with low mortality; MSAP can cause transient organ failure (duration < 48 h) combined with local or systemic complications; SAP is manifested by persistent organ failure (duration > 48 h) or death with generally poor prognosis. Therefore, early diagnosis and accurate assessment of disease severity is of great importance to improve the management of AP. Currently, several scoring systems have been applied to predict AP outcome. Ranson's criteria [5] are probably the most used scoring system but it can only be determined after 48 hours of inpatient observation. The Acute Physiology and Chronic Health Evaluation II (APACHE II) scoring system can provide effective monitoring of the patients during the whole course but is very complex to use [6]. While laboratory indexes such as IL-6, serum amylase, and lipase has relative high feasibility, the result has been inconsistent and has been shown to be unrelated to the severity of the disease [7, 8]. In the present study, we retrospectively assessed the clinical and laboratory data of 166 AP patients hospitalized in Baotou Central Hospital in China and aim to investigate the early indicators that predict the mortality of AP.

## 2. Materials and Methods

### 2.1. Patients

In total, 166 AP patients were hospitalized in the General Surgery Department and Gastroenterology Department of Baotou Central Hospital, China, between January 2014 and November 2015. The clinical data were retrospectively collected from medical records and examination results of the patients. Pancreatitis is diagnosed based on the revised Atlanta classification [4]: serum amylase levels increased more than three times of the normal threshold value (120 U/l) and/or peculiar findings in abdominal computed tomography (CT) or abdominal ultrasound results. Patients with pancreatitis postendoscopic retrograde cholangiopancreatography, suspected malignant biliary and pancreatic diseases, nonpancreatic infection or infection caused by other diseases, and pancreatitis diagnosed in surgery as well as patients with history of immunodeficiency were excluded from the study. The study was registered and approved by the ethics board of Baotou Central Hospital. This is an analysis of retrospective data and no patient's identifiable information was used in the analysis; the written informed consent form was waived for this study.

### 2.2. Data Collection

Venous blood of each patient was collected for laboratory examinations using SYSMEX5000 hematology analyzer and Hitachi 7600 analyzer within 24 hours after admission. The parameters included alanine aminotransferase (ALT), aspartate aminotransferase (AST), total bilirubin (TBIL), direct bilirubin (DBIL), indirect bilirubin (IBIL), total protein, albumin, glucose, urea nitrogen, creatinine, creatine kinase, amylase, lipase, calcium, white cell count (WCC), red blood cell (RBC), hemoglobin, hematocrit, mean corpuscular volume (MCV), platelet, neutral cell ratio (NCR), lymphocyte ratio (LYR), red cell distribution width (RDW), and urine amylase (UAMY).

### 2.3. Statistics

Data with normal and nonnormal distribution were presented as mean ± SD and median with range, respectively. *χ*^2^ test was used to compare the sex distribution between survivor and nonsurvivor. Student's *t*-test, Kruskal-Wallis H test, and Kolmogorov-Smirnov *Z* test were used to perform univariate analysis. Variables showing significant difference between survivors and nonsurvivors in univariate analysis were included in multivariate logistic regression analysis. Receiver operating characteristic (ROC) curves were drawn to indicate the prognostic values of every selected indicator for AP mortality. The probability of the cut-off values was calculated adopting the Youden index, which is based on the optimal combination of sensitivity and specificity. All statistical analysis was performed using SPSS V.16.0 software. *P* value <0.05 were considered to be statistically significant.

## 3. Results

**Figure 1:**
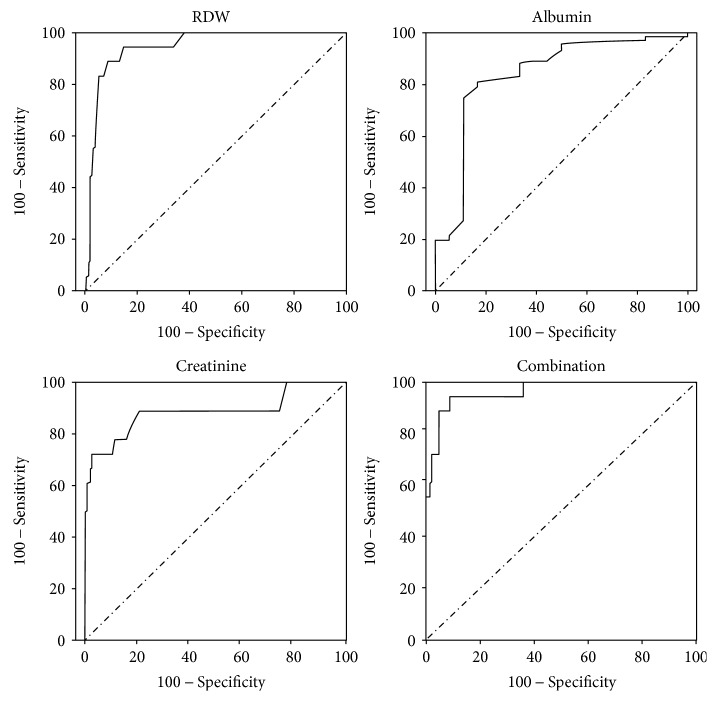
ROC curves for red cell distribution (RDW), creatinine, albumin, and the combination of the three indicators (combination).

In total, 166 patients with AP (104 males and 62 females; median age 53.7, range 24–79) were selected for the present study. Based on the Atlanta classification, the number of patients diagnosed as MAP, MSAP, and SAP was 83 (50.0%), 42 (25.3%) and 41 (24.7%), respectively, with a corresponding mean hospital stay of 11.1, 13.0, and 17.6 days. The etiology of AP was biliary in 67 patients (40.4%), hyperlipidemic in 39 patients (23.5%), alcoholic in 11 patients (6.6%), and other causes in 49 patients (29.5%). There were 18 death events: 6 cases were caused by septic and toxic shock, 5 cases by multiple organ dysfunction syndrome, 4 cases by disseminated intravascular coagulation, 2 cases by acute renal insufficiency, and 1 case by respiratory and cardiac arrest. Among the 148 survivors, 10 patients had local pancreatic complications when they were discharged from the hospital, including 8 with pancreatic pseudocyst and 2 with pancreatic abscess. Detailed clinical characteristics and laboratory indexes in AP patients stratified by survivors and nonsurvivors were shown in Table 1. We first performed univariate analysis to identify the potential laboratory indexes that are associated with the mortality of AP. We found that AP nonsurvivors had significantly increased levels of AST, urea nitrogen, creatinine, creatine kinase, MCV, and RDW but decreased levels of albumin, calcium, RBC, hemoglobin, and hematocrit (all *P* < 0.05, Table 1). Next, we performed multivariate logistic regression analysis and included the indicators (AST, urea nitrogen, creatinine, creatine kinase, MCV, RDW, albumin, calcium, RBC, hemoglobin, and hematocrit) that showed a significant difference in the univariate analysis. We found that RDW and creatinine significantly increased the risk of AP mortality; in contrast, albumin was shown as a protective factor (RDW: *P* = 0.001, OR = 2.97, 95% CI for OR: 1.54–5.72; creatinine: *P* = 0.005, OR = 1.03, 95% CI for OR: 1.01–1.04; albumin: *P* = 0.032, OR = 0.92, 95% CI for OR: 0.85–0.99). Further, we examined the effectiveness of RDW, creatinine, and albumin in the prediction of AP mortality using ROC analysis (Figure 1). The area under curve (AUC) value of these three variables was statistically significant to predict AP mortality. RDW had the highest sensitivity (88.9%) and albumin had the highest specificity (97.3%). Detailed results were shown in Table 2. The AUC of the combined measurement of RDW, creatinine, and albumin was 0.964 (*P* < 0.001, 95% CI: 0.92 to 1.00) with RDW ≥ 14.45%, creatinine ≥ 125.5 *μ*mol/l, and albumin ≤ 34.95 g/l indicating a good predictive value for mortality among patients with AP. The combined measurement had a sensitivity of 100% and specificity of 64.2% (Figure 1).

## 4. Discussion

AP is a serious acute abdomen disease which can lead 10–20% of the patients to develop a severe course including pancreatic necrosis, multiple organ dysfunction syndrome, infectious complications, and other adverse consequences [9]. In this study, we retrospectively evaluated the serological indicators that are associated with the mortality of AP in 166 patients. We found increased levels of RDW and creatinine but decreased levels of albumin in AP nonsurvivors compared to the survivors, suggesting RDW and creatinine may contribute a risk while albumin as a protective factor in AP mortality.

RDW is a parameter obtained from the hematology analyzer, indicating the heterogeneity of the circulating erythrocytes. It is calculated by dividing the histogram width of 68.26% of RBCs by the mean corpuscular volume; the result of which was then multiplied by 100. The rise of RDW reflects variable volume enlargement of red blood cells, which was associated with a number of diseases including coronary artery disease, stroke, renal insufficiency, and severe infection [10–12]. The increasing number of studies has shown that increased RDW is associated with mortality in AP [13–16]. It is surprising to find RDW as a prognostic factor of AP mortality for many researchers who thought that we have known plenty of the complete blood count and specifically RDW. However, when Horne conducted a study to adjust a multiplicity of covariables (including demographics, anthropometrics, diagnoses and comorbidities, laboratory variables, behavioral factors, nutritional parameters, and medical treatments) that may affect the association of RDW with adverse events, the predictive effect of RDW still remains [17]. In our study, we found that the RDW value in AP nonsurvivors (15.34%) is significantly higher than that in AP survivors (13.35%). Although the pathophysiological mechanism of RDW and AP mortality is unclear, several mechanisms have been proposed. Inflammatory mediators play an important role in the progression of the disease in patients with AP, which is suggested as the trigger to alter the channels of glycoprotein and ion on the surface of the cytomembrane of the red blood cells, resulting in the changes of the red cell morphology [18]. Besides, the oxidative pressure was shown to affect the deformability and the half-life circulation of red cells. It can also affect the survival time of red cells by destruction of nucleic acids, proteins, and lipids, which results in the immature red cells entering into the peripheral blood circulation and thus increasing RDW [19]. Our study has meaningful clinical implications. Daily monitoring of RDW is a reliable and inexpensive practice which will not increase the cost of traditional complete blood count since this parameter is automatically generated by all modern hemocytometers. Therefore, tightened clinical management should be given to patients exhibiting a significant increase of RDW during hospitalization, especially those displaying early increase during the first days of hospital stay.

Muddana et al. suggested that a serum creatinine value > 159 *μ*mol/l at 48 hours indicated a higher possibility of the occurrence of pancreatic necrosis (PN), which is a major contributing factor to morbidity and mortality of AP, especially when infection occurs [20]. Consistently, Lipinski et al. found that creatinine levels were significantly increased in AP with PN both on admission and 48 h later, and the measurement on the first day proved to be a good predictor of mortality [21]. We found that AP nonsurvivors had significantly higher levels of creatinine (281.28 ± 253.15 *μ*mol/l) compared to the survivors (63.33 ± 23.72), which was in line with Muddana et al.'s finding. However, in a prospective multicenter study by Lankisch et al., the author suggested the low sensitivity of creatinine in predicting pancreatic necrosis which requires further investigation [22]. PN is a complex pathogenesis. Active pancreatic inflammation with increased vascular permeability, vascular spasm, shunting of the blood from visceral organs, and increased viscosity of hemoconcentrated blood may all contribute to regional pancreatic infarctions [23]. Increased creatinine levels are markers of renal dysfunction, which suggest intravascular hypovolemia if presented as acute changes at the baseline. We speculated that creatinine may be less sensitive to small intravascular volume changes and can better reflect visceral organ injury. Based on our results, when AP patients present high levels of RDW and creatine on the first day, special consideration should be taken since these patients may bare higher risk of disease mortality.

It is generally believed that the occurrence of AP can cause damage in liver function, resulting in reduced synthesis of serum albumin. Besides, effusion in the pancreas and its surrounding tissue in the presence of AP will lead to excessive loss of albumin. Elastase secreted from the pancreas and neutrophils can also promote the effusion of albumin through the damaged vessel wall. Thus, albumin levels may decrease sharply at the early stage of AP. Tannuri et al. indicated that reduction in serum albumin level was observed in critically ill patients. When serum albumin < 30.3 g/l, the risk of mechanical ventilation will double, the stay will be longer, and the mortality will be higher as compared with that of the patients with normal albumin levels [24]. In contrast, the remedy success rate will double as the serum albumin level increase by every 5 g/l. Thus, serum albumin is regarded as an independent risk factor for the mortality of patients with critical diseases and one of the prognosis indicators at admission [25]. Although there is not any report suggesting albumin as an independent risk factor for the mortality of AP, a recent study suggested that C-reactive protein/albumin ratio > 16.28 had a 92.1% sensitivity and 58.0% specificity in predicting AP mortality [26]. We found that the serum albumin level in nonsurvivor group was significantly lower than that in survivor group. The optimal cut-off value for albumin was 34.95 (sensitivity = 87.8%, specificity = 66.7%), and the prediction percentage of the mortality of AP patients could reach 54.5%.

There are few limitations in our current study: this is a retrospective study; thus, it is difficult to draw a causal conclusion. We did not have the follow-up data of RDW, creatinine, and albumin changes in AP survivors when they were discharged; thus, we could not infer a long-term association of AP mortality. We should recognize that creatine and albumin levels have significant limitations as we identified a relatively low specificity of creatinine (66.7%) and low sensitivity of albumin (72.2%). This is a single center research although we have a relatively large sample size.

In conclusion, our results suggested that serological indicators RDW, creatinine, and albumin levels examined at hospital admission were associated with AP mortality. The underlying mechanisms require further clinical investigation.

## Figures and Tables

**Table 1:** Clinical characteristics and laboratory data of survivors and nonsurvivors of AP.

	Nonsurvivors (*n* = 18)	Survivors (*n* = 148)	*P* value
Age (years)	57.56 ± 15.36	53.20 ± 17.17	0.306
Gender (% male)	83.33	60.14	0.055
ALT (U/l)	38.5 (7–2282)	43 (7–704)	0.985
AST (U/l)	92 (9–7723)	33 (7–882)	0.006^∗∗^
TBIL (*μ*mol/l)	23.6 (3.8–125.4)	18.5 (2.5–1936)	0.683
DBIL (*μ*mol/l)	9.25 (2–95.3)	6.35 (0.2–270)	0.189
IBIL (*μ*mol/l)	11.20 ± 6.75	14.01 ± 8.60	0.184
Total protein (g/l)	60.46 ± 12.45	65.73 ± 10.50	0.050
Albumin (g/l)	31.87 ± 8.84	42.00 ± 7.42	<0.001^∗∗∗^
Glucose (mmol/l)	9.31 ± 4.97	9.02 ± 4.93	0.819
Urea nitrogen (mmol/l)	16.46 ± 14.16	5.89 ± 5.16	<0.001^∗∗∗^
Creatinine (*μ*mol/l)	281.28 ± 253.15	63.33 ± 23.72	<0.001^∗∗∗^
Creatine kinase (U/l)	1780.67 ± 4949.34	85.21 ± 64.21	<0.001^∗∗∗^
Amylase (U/l)	383 (18.4–3928)	201.6 (1.19–3714)	0.110
Lipase (U/l)	329.35 (15.3–10,218)	336.5 (1.23–6356.8)	0.884
Calcium (mmol/l)	1.96 ± 0.36	2.30 ± 0.28	<0.001^∗∗∗^
WCC (mm^3^)	14.31 ± 5.8	12.51 ± 5.82	0.219
RBC (mm^3^)	4.05 ± 1.18	4.85 ± 0.77	<0.001^∗∗∗^
Hemoglobin (g/l)	121.28 ± 44.77	151.07 ± 22.25	<0.001^∗∗∗^
Hematocrit (%)	35.95 ± 13.42	43.10 ± 9.17	0.004^∗∗^
MCV (fl)	94.49 ± 5.76	91.66 ± 5.28	0.035^∗^
Platelet (10^9^/l)	168.83 ± 128.31	202.47 ± 72.28	0.094
NCR (%)	83.91 ± 8.78	81.24 ± 10.77	0.314
LYR (%)	9.13 ± 6.72	12.18 ± 8.74	0.155
RDW (%)	15.34 ± 0.97	13.35 ± 0.92	<0.001^∗∗∗^
UAMY (U/l)	1592.05 (106.8–31452)	1522 (31–32212)	0.661

ALT: alanine aminotransferase; AST: aspartate aminotransferase; TBIL: total bilirubin; DBIL: direct bilirubin; IBIL: indirect bilirubin; WCC: white cell count; RBC: red blood cell; MCV: mean corpuscular volume; NCR: neutral cell ratio; LYR: lymphocyte ratio; RDW: red cell distribution width; UAMY: urine amylase. ^∗^*P* < 0.05, ^∗∗^*P* < 0.01, and ^∗∗∗^*P* < 0.001.

**Table 2:** ROC analysis for biochemical parameters of patients with acute pancreatitis.

Parameters	AUC (95% CI)	*P* value	Cut-off	Sensitivity (%)	Specificity (%)	PPV (%)
RDW (%)	0.92 (0.73–0.96)	<0.001^∗∗∗^	14.45	88.9	91.2	80.1
Creatinine (*μ*mol/l)	0.88 (0.77–1.00)	<0.001^∗∗∗^	34.95	87.8	66.7	54.5
Albumin (g/l)	0.84 (0.90–0.99)	<0.001^∗∗∗^	125.5	72.2	97.3	69.5

RDW: red cell distribution width; AUC: area under the curve; PPV; positive predictive value. ^∗∗∗^*P* < 0.001.
